# Interpretation and Natural History of Asymmetric Skin Folds in Infants With Developmental Dysplasia of the Hip

**DOI:** 10.7759/cureus.64926

**Published:** 2024-07-19

**Authors:** Panagiotis V Samelis, Evmorfia Pechlivanidou, George Vasileiou, Dimitrios Artsitas, Panagiotis Kolovos

**Affiliations:** 1 Orthopaedics, Apostolos Pavlos Trauma Hospital, Athens, GRC; 2 Orthopaedics, Orthopaedic Research and Education Center, Attikon University Hospital, Athens, GRC; 3 Orthopaedics, Panagiotis & Aglaia Kyriakou Children's Hospital, Athens, GRC; 4 Hygiene, Epidemiology, and Medical Statistics, National and Kapodistrian University of Athens School of Medicine, Athens, GRC

**Keywords:** galleazi sign, asymmetric skin fold, asymmetric skin crest, asc, developmental dysplasia of the hip, ortolani hip, asf, ddh

## Abstract

The association between asymmetric skin folds (ASFs) of the gluteal, groin, or thigh regions and ipsilateral developmental dysplasia of the hip (DDH) has not been elucidated yet. Why are ASFs formed in some infants with DDH? Do DDH-associated ASFs persist during childhood and adulthood? Is it possible for ASFs to emerge without DDH pathology? Three cases of acute and chronic hip pathology in adults are presented in an attempt to explain the formation and the natural history of ASFs in infants with DDH. It is suggested that ASFs are formed when the excess soft tissues of the thigh shrink over a short femur. On the other hand, ASFs disappear after the length of the thigh is restored and the soft tissues of the thigh are re-stretched. This telescoping mechanism of the formation and disappearance of ASFs is applicable regardless of the underlying hip pathology or the age of the patient.

## Introduction

Developmental dysplasia of the hip (DDH) is a frequent musculoskeletal disorder in infants [1]. The spectrum of DDH is broad, ranging from stable dysplasia to complete hip dislocation [2]. Early detection and treatment are mandatory to provide a healthy hip and to avoid early-onset hip osteoarthritis and total hip replacement in young adults [3]. Detection of DDH is based on history, clinical examination, and, for a few decades, ultrasound examination of the hips of the newborn [3-5]. A history of breech presentation, swaddling, and positive family history, along with clinical findings such as limited hip abduction, asymmetric skin folds (ASFs), limb length discrepancy, and hip instability, increase the suspicion for DDH and prompt further investigation of the newborn [2]. Hip ultrasound provides significant information about hip morphology and stability without infant irradiation. Furthermore, it is applicable in the immediate postnatal period. On the other hand, it has to be performed and evaluated by an experienced examiner to avoid overdiagnosis and overtreatment [3].

In the past, isolated ASFs of the gluteal, groin, or thigh regions were deemed significant for the detection of DDH of the infantile hip [2, 6, 7]. Even today, this sign is the most common indication for DDH referral among general practitioners [8]. Based on the acetabular index of the alpha angle, Louer et al. found that 79% of infants referred for isolated ASFs had acetabular dysplasia, and 38% received treatment by an orthosis [9]. Omeroglu et al. report that the risk of sonographically diagnosed DDH increases by 3.5 times in the presence of isolated ASFs [10]. On the other hand, several studies renounced ASFs as a screening test for DDH because most infants with ASFs do not present DDH [11-13].

Nevertheless, the presence of ASFs in infants already diagnosed with DDH provides significant information and deserves further attention. Which mechanism creates ASFs? What do ASFs tell us about DDH progression? What is the fate of the ASFs with the ongoing growth of the limb? Do DDH-associated ASFs of the infant persist in adulthood? Is it possible for ASFs to emerge in non-DDH adults secondary to acquired hip pathology? Three cases of hip pathology in adults are presented in an attempt to explain the formation and the natural history of ASFs.

## Case presentation

**Figure 1:**
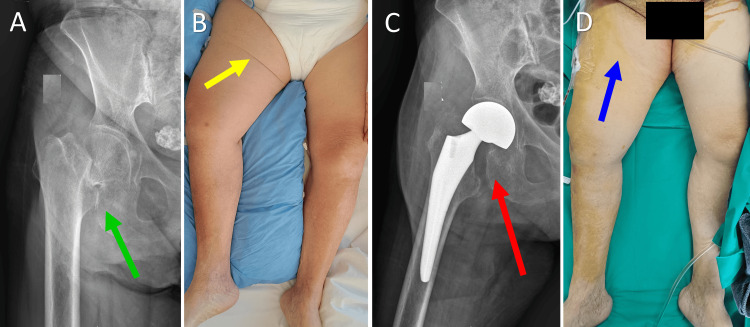
Asymmetric skin fold (ASF) formation in a 76-year-old woman after a femoral neck fracture A. An anteroposterior pelvic X-ray indicating the femoral neck fracture of the right hip (green arrow); B. The ASF formation on the right thigh is due to proximal migration of the right femur and subsequent limb shortening (yellow arrow). C. An anteroposterior X-ray indicating a hemiarthroplasty of the right hip (red arrow); C. The ASF disappeared after fracture treatment and restoration of limb length (blue arrow). Image credits: P. Samelis

The first case is a 76-year-old female patient with a subcapital fracture of the right hip. On inspection, external rotation and acute shortening of 3 cm of the affected limb were manifested. A medial transverse skin fold was evident preoperatively on the anteromedial proximal thigh, which was not present on the contralateral healthy limb. The patient underwent a hemiarthroplasty of the right hip three days after admission. Hip hemiarthroplasty restored limb length, and, surprisingly, the ASF disappeared immediately while the patient was still in the operating theater (Figure 1).

**Figure 2:**
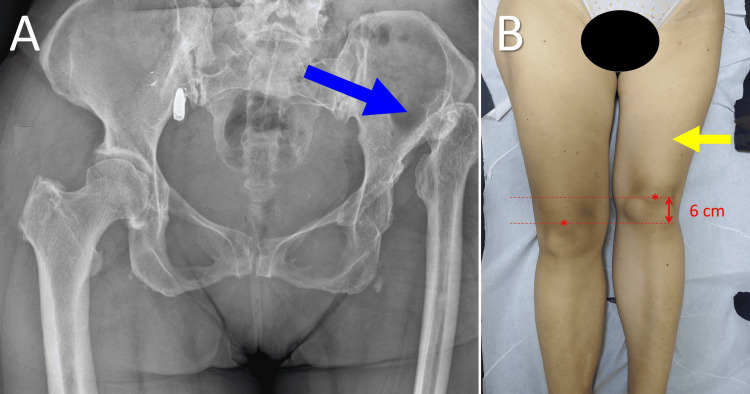
Neglected high dislocation of the left hip in a 52-year-old woman A. An anteroposterior X-ray of the pelvis indicating high dislocation of the left hip with a false acetabulum (blue arrow); B. No asymmetric skin fold is evident on the 6 cm shorter left thigh (yellow arrow; asterisks indicate the center of the patellae). Image credits: P. Samelis

The second case is a 52-year-old female with a painful limp and a 6 cm shorter left limb. Limb length discrepancy (LLD) has existed since infancy. Radiologic examination revealed a neglected high dislocation of the left hip, type-C1, according to the Hartofilakidis classification of adult DDH [14]. No ASFs were detected on inspection of the left thigh, the groin, or the gluteal region. The skin on both thighs of the patient was smooth, in spite of the significant but chronic, life-long shortening of the left lower limb. A total hip replacement was scheduled (Figure 2).

**Figure 3:**
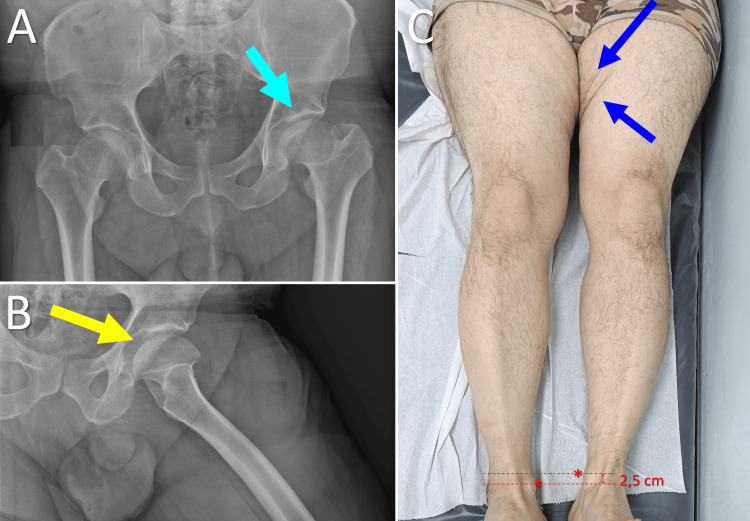
Asymmetric skin fold (ASF) formation in a 44-year-old patient with growth disturbance of the proximal femoral epiphysis and limb length inequality Α. Anteroposterior pelvic X-ray indicating bilateral growth disturbance of the proximal femur, more prominent on the left side (light blue arrow); B. Morphologic changes of the proximal femur are evident on the lateral x-ray view of the left hip (yellow arrow); C. Two ASFs (blue arrows) are formed on the 2.5 cm shorter left thigh (asterisks indicate the medial malleoli). Image credits: P. Samelis

The third case is that of a 44-year-old male, with two ASFs on the medial side of the left thigh and a shorter left lower leg of about 2.5 cm. The patient was admitted to the hospital after a car accident. He had a history of marked obesity (BMI = 46.2 kg/m^2^), dating from late childhood and adolescence. The patient did not recall a painful limp during childhood and adolescence, nor did he consult any pediatrician for obesity. Five years ago, the patient had lost about 50 kg of weight secondary to bariatric surgery by means of a gastric sleeve. On the anteroposterior pelvic X-ray, bilateral coxa magna and coxa breva were evident, more prominent on the left side. The acetabulum was normal, without evidence of dysplasia or early arthrosis of both hips. The articulo-trochanteric distance (the distance between the tip of the greater trochanter and the top of the femoral head) was lower on the left side compared to the right side, which was the main cause of LLD. The underlying pathology of the hips was probably a bilateral Perthes disease or an endocrine-disease-dependent growth disturbance in late childhood and adolescence. Unfortunately, the patient could not provide an accurate history or X-rays at this age. However, it seems that femoral head remodeling progressed quite well, and the patient is now able to perform his daily activities without a painful limp (Figure 3).

## Discussion

**Figure 4:**
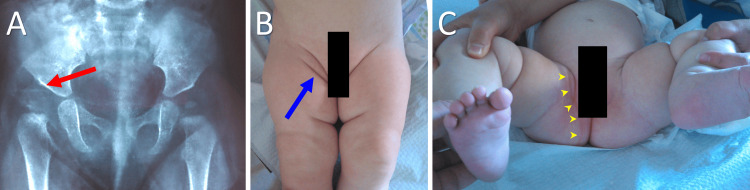
Asymmetric skin fold (ASF) formation in a seven-month-old infant with a right Ortolani hip A. Anteroposterior pelvis projection indicating a dislocated dysplastic right hip (red arrow); B. Inguinal ASF formation ipsilateral to the dislocated hip (blue arrow); C. The ASF extends deep into the buttock area (arrowheads). Image credits: P. Samelis

**Figure 5:**
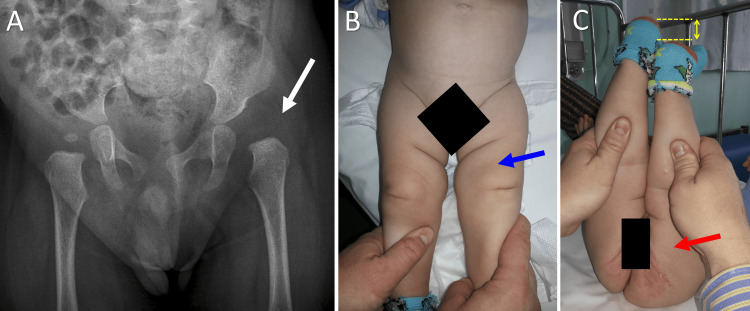
No asymmetric skin fold (ASF) formation seen in a six-month-old infant with a left Ortolani hip A. An anteroposterior pelvic view of the X-ray indicating dislocation of the left hip (white arrow); B. No ASF is detected on the left thigh or groin (blue arrow); C. Proximal migration of the left femur is evident, leading to a shorter left thigh (yellow arrow, yellow lines). No ASFs are evident on the left gluteal region as well (red arrow). Image credits: P. Samelis

**Figure 6:**
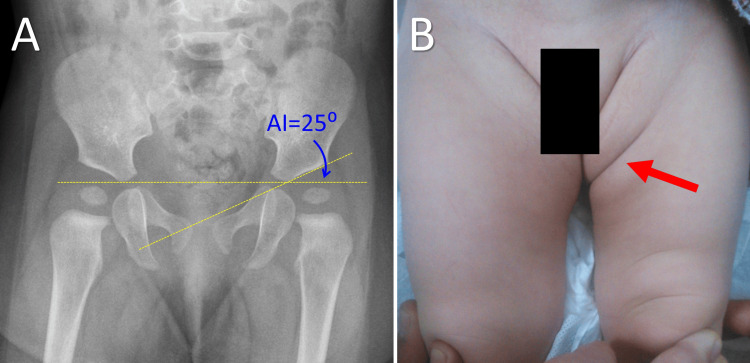
Asymmetric skin fold (ASF) formation in a five-month-old infant with healthy hips A. An anteroposterior X-ray projection of the pelvis. No signs of dysplasia of the left hip are evident. The acetabular (AI) index is 25 degrees; B. A huge, deep ASF is manifested on the left thigh (red arrow). Image credits: P. Samelis

Infants with DDH may or may not present ASFs (Figures 4, 5). Omeroglu et al. found that ASFs were present in only 26% of infants who had sonographically diagnosed DDH according to the Graf method [10]. On the other hand, ASFs may be a normal variation in up to 25% of healthy infants (Figure 6) [13]. It has been reported that ASFs, especially inguinal folds, are more likely to be observed in DDH with hip subluxation or dislocation [2, 7], implying that ASF formation may be associated with higher degrees of dysplasia. 

Based on the presented cases, a hypothesis on ASF formation and disappearance relative to hip pathology is expressed. This theory might apply to ASFs related to DDH.

**Figure 7:**
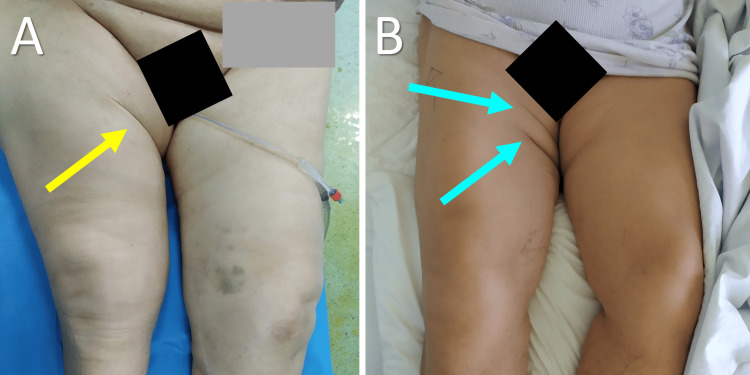
Asymmetric skin fold (ASF) formation after acquired hip pathology A. ASF formation after periprosthetic fracture of the right hip in a 90-year-old patient (yellow arrow); B. Multiple ASF formation after prosthetic joint dislocation of the right hip in a 78-year-old patient (blue arrows). Image credits: P. Samelis

It is suggested that ASFs are formed when the soft tissue envelope around the femur, especially the skin and subcutaneous fat, shrinks over a shortened thigh, as is the case of the patient with the subcapital hip fracture. This hypothesis is further supported by the disappearance of the ASF immediately after fracture treatment, which restored the original limb length. Asymmetric skin folds are always located on the medial side because limb shortening secondary to hip pathology is usually accompanied by adduction of the lower limb. Since this first patient, we have noticed ipsilateral ASFs in several consecutive patients with limb shortening secondary to acquired hip pathology, such as a prosthetic hip joint dislocation, a Girdlestone procedure, or a periprosthetic hip fracture. In the cases that were treated, the ASF disappeared or was mitigated after fracture treatment and limb length restoration (Figure 7).

The absence of ASFs in the patient with untreated dislocation deserves further explanation. This patient was never diagnosed with DDH in infancy. According to our hypothesis, in the DDH infant, the evolution of a dysplastic to a permanently subluxed or a resting dislocated (Ortolani) hip results in thigh shortening due to proximal migration of the femoral head (Galleazzi sign). This leads to a relative surplus of the soft tissues around the femur. A recently dislocated hip is not expected to present an ASF if prompt detection and reduction occur. However, if the reduction is delayed, the shrunk soft tissues around the apparently shortened femur form an ASF. Through the same mechanism, an ASF is created in the adult after acute femur shortening following a femoral neck fracture. It is not known how many days it takes for the ASF to emerge; however, an ASF in the DDH infant may indicate the progression of stable dysplasia to subluxation or dislocation and, thus, delayed diagnosis and treatment. Consequently, the presence of ASFs in an infant with hip dysplasia may be associated with a worse prognosis compared with an ASF-free DDH infant. Treatment aims to reduce and stabilize the hip, while further delay of treatment results in a fixed hip dislocation. In the growing infant, either stable reduction or fixed dislocation reversed the process, which formed the ASF. The shrunk soft tissues of the thigh are re-stretched either acutely (hip reduction) or gradually (axial growth of the femur), and the ASF disappears with time. Thus, ASFs are not expected in the adult with untreated DDH, in spite of the marked persisting LLD, as shown for the second patient. 

The question is, why did the third patient still present an ASF? Does this fit with the suggested mechanism for the creation and disappearance of ASFs? The answer is that this patient confirms the theory. After hip pathology occurred, the progression of the disease led to the gradual shortening of the femoral bone in relation to the obese soft tissue envelope of the thigh. Thus, ASFs, possibly bilaterally, were gradually formed as the femoral head-neck deformity progressed and both femurs shortened (in this case, the term symmetric skin folds would be a more suitable description). Growth disturbance and subsequent thigh shortening on the right side were less severe compared to the left side, so the remaining growth of the right femur was effective in eliminating the (hypothetical) ASF on the right side until skeletal maturity was reached. On the other hand, the remaining growth did not suffice to stretch the soft tissues on the left side. Thus, the ASF on the left side remained beyond adolescence. The difference in appearance between the two thighs was probably exacerbated after the recent marked weight loss of the patient.

To our knowledge, a pathologic mechanism that links ASF formation with DDH progression has not been described yet. The proposed mechanism helps to appreciate the significance of ASFs in the DDH-infant. Thus, ASFs may not be useful for screening for DDH; however, they are a very important clinical sign. The presence of ASFs in the DDH infant indicates that an originally normal limb, relative to its length, starts to shorten due to proximal migration of the femoral head out of the acetabulum. Limb shortening parallels the progression of hip dysplasia towards subluxation and complete hip dislocation. If the rate of limb shortening is higher than the rate of axial growth of the femur, a relatively soft tissue surplus is created around the apparently shorter femur. The soft tissues shrink and create ASFs. In summary, the presence of ASFs in the DDH infant reflects the progression, severity, and chronicity of DDH and may be associated with a worse prognosis and irreversible, by means of conservative treatment, late dysplasia.

## Conclusions

The formation of ASFs is a purely mechanical process. Asymmetric skin folds emerge when the soft tissues of the thigh shrink over a shortened femur. This telescoping effect of the soft tissues may create ipsilateral ASFs, regardless of the underlying etiology of femur shortening. Thus, ASFs may emerge in adults or adolescents after acquired hip pathology or in infants with a recently established hip subluxation or dislocation secondary to DDH. Asymmetric skin folds disappear or are mitigated after restoration of limb length, either after treatment of the hip pathology of the adult or by hip reduction and/or limb growth in the infant with DDH. Especially in the DDH infant, the presence of ASFs may indicate progression and late detection of dysplasia.
